# Improving virus production through quasispecies genomic selection and molecular breeding

**DOI:** 10.1038/srep35962

**Published:** 2016-11-03

**Authors:** Francisco J. Pérez-Rodríguez, Lucía D’Andrea, Montserrat de Castellarnau, Maria Isabel Costafreda, Susana Guix, Enric Ribes, Josep Quer, Josep Gregori, Albert Bosch, Rosa M. Pintó

**Affiliations:** 1 Enteric Virus Laboratory, Department of Genetics, Microbiology and Statistics, School of Biology, University of Barcelona, Barcelona, Spain; 2 Enteric Virus Laboratory, Institute of Nutrition and Food Safety, Campus Torribera, University of Barcelona, Santa Coloma de Gramenet, Spain; 3 Enteric Virus Laboratory, Department of Cell Biology, Physiology and Immunology, School of Biology, University of Barcelona, Barcelona, Spain; 4 Liver Unit. Internal Medicine. Lab. Malalties Hepàtiques. Vall d’Hebron Institut Recerca-Hospital Universitari Vall d’Hebron (VHIR-HUVH), Barcelona, Spain; 5 Centro de Investigación Biomédica en Red (CIBER) de Enfermedades Hepáticas y Digestivas (CIBERehd) del Instituto de Salud Carlos III, Spain.; 6 Roche Diagnostics SL, Barcelona, Spain

## Abstract

Virus production still is a challenging issue in antigen manufacture, particularly with slow-growing viruses. Deep-sequencing of genomic regions indicative of efficient replication may be used to identify high-fitness minority individuals suppressed by the ensemble of mutants in a virus quasispecies. Molecular breeding of quasispecies containing colonizer individuals, under regimes allowing more than one replicative cycle, is a strategy to select the fittest competitors among the colonizers. A slow-growing cell culture-adapted hepatitis A virus strain was employed as a model for this strategy. Using genomic selection in two regions predictive of efficient translation, the internal ribosome entry site and the VP1-coding region, high-fitness minority colonizer individuals were identified in a population adapted to conditions of artificially-induced cellular transcription shut-off. Molecular breeding of this population with a second one, also adapted to transcription shut-off and showing an overall colonizer phenotype, allowed the selection of a fast-growing population of great biotechnological potential.

Virus production remains a critical point for antigen manufacturing especially when large amounts of antigens are needed for inactivated vaccines or diagnostic kits. A clear example is the production of hepatitis A virus (HAV), a very challenging virus to grow^1^, with only a few strains adapted to replicate *in vitro*. Large scale-ups are required to produce industrial batches resulting in very high costs.

The slow replication phenotype of HAV may be explained by three interrelated key factors. First an internal ribosome entry site (IRES) very inefficient in directing translation^2^,^3^, second the inability of the virus to induce the cellular protein synthesis shut-off^4^ and third a highly deoptimized codon usage^5^,^6^. Although recently synthetic biology has proven highly effective in recoding viral RNA genomes^7^, when there is an interplay between codon usage and other genetic features as above mentioned, recoding strategies are much harder to anticipate. An alternative is to molecularly breed genomically selected parental types. Genomic selection is based on the use of genomic information to predict breeding values for particular phenotypes without knowledge of the individual genes contributing to them^8^,^9^,^10^. Genomic selection has been used in livestock breeding, particularly in dairy cattle^11^. The genome size of cattle (*Bos taurus*) is 3.7 × 10^5^ times higher than that of a *Picornaviridae* member. However, the short generation times and the high mutation rates of RNA viruses result in complex virus populations termed quasispecies^12^, which may contain over 10^7^ individuals. Consequently, while genomic selection in cattle relies on whole genome sequences from single individuals, in virus populations deep-sequencing of certain genome regions unraveling the individual composition may be predictive as well. With the aim to select a HAV fast-growing population we adapted the strain HM175 43c to conditions of low and high artificially-induced transcription shut-off in FRhK-4 cells, and two populations, F0.05LA and F0.2LA, showing changes in capsid codon usage were selected^13^. Transcription shut-off was achieved with actinomycin D (AMD), which specifically inhibits cellular DNA-dependent RNA polymerases with no effect on the viral RNA-dependent RNA polymerase. Under these conditions of lower cellular protein synthesis, the virus evolved by changing its codon usage^13^. In the present study, deep-sequencing of two genome fragments (belonging to the IRES and the VP1 regions) was used to predict “breeding values” of individuals present in these populations, based on mutations affecting IRES activity and translation efficiency, respectively. A minority of individuals in population F0.05LA was predicted to have a high “breeding value” defined as a fast replication and productive phenotype.

Virus populations may contain competitor and colonizer individuals^14^. In very simple words competitors and colonizers may be defined as those viruses which in coinfections show advantage over others intracellularly (i.e. outcompeting resources) or extracellularly (i.e. more efficient spread), respectively. Whether competitors or colonizers will outcompete each other in a coinfection depends on the multiplicity of infection (MOI) and on their frequencies^14^,^15^,^16^,^17^. A low MOI, in which a fraction of cells remains uninfected, together with a threshold level of viruses are required for a colonizer to outcompete a competitor. In contrast, a high MOI is advantageous for a competitor to outcompete a colonizer. Population F0.05LA was predicted to contain very low-frequency high-fitness individuals suppressed by the quasispecies, and whose isolation by simply playing with MOI or population size was anticipated to be difficult^15^,^17^. These high-fitness individuals were predicted to be good colonizers (faster than the suppressors due to an efficient IRES) and also quite good competitors (more optimized codon usage than many of the suppressors). We took advantage of population F0.2LA which has a colonizer behavior in competition experiments with population F0.05LA under conditions of low cellular shut-off and low MOI^6^. This behavior is mostly related to its very efficient uncoating process^13^, which ensures a much faster cellular cycle. Molecular breeding of populations F0.05LA and F0.2LA was performed to induce a disturbance in the quasispecies, which may help to rescue the minor fraction of individuals with potentially high breeding values. In doing so, a fast-growing population producing larger plaques and higher virus titers was selected, with a remarkable biotechnological added value.

## Results

### Genome regions potentially predictive of the breeding values of quasispecies individuals

**Figure 1:**
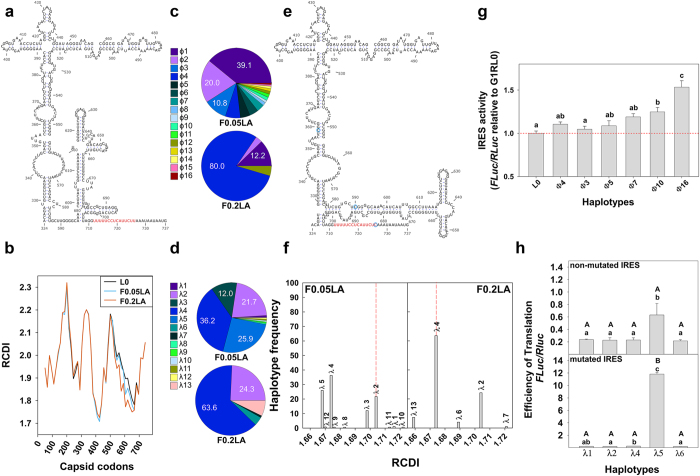
Genomic selection based on fragments belonging to IRES and VP1 regions. (**a**) Predicted secondary structure of the HAV IRES. In red the second polypyrimidine tract (pY2). (**b**) RCDI of the capsid-coding region. (**c**) Percent of haplotypes detected in the IRES fragment of populations F0.05LA and F0.2LA. (**d**) Percent of haplotypes detected in the VP1 fragment of populations F0.05LA and F0.2LA. (**e**) Alternative secondary structure predicted in several IRES haplotypes. In red pY2. Blue circles indicate the replacements found in ɸ16 haplotype. (**f**) RCDI values of the VP1 haplotypes. Red line corresponds to the haplotype whose sequence coincides with the consensus. (**g**) IRES activity figured as the mean and standard error of the FLuc/RLuc ratio of different haplotype-derived vectors compared to the ancestor L0 type. Three different experiments, including two replicas each, for each haplotype were performed. Statistically significant differences (P < 0.05; Student’s t-test) between haplotypes are indicated by different letters: a ≠ b, a ≠ c, b ≠ c, ab = a, ab = b, ab ≠ c. (**h**) Translation efficiency of different VP1 haplotype-derived vectors under the control of the wild type IRES without or with mutations U359C, U590C and U726C. Values represent the mean and standard error of three different experiments, including two replicas each, for each haplotype. Statistically significant differences (P < 0.05; Student’s t-test) between haplotypes are indicated by different letters: a ≠ b, a ≠ c, b ≠ c, ab = a, ab = b, ab ≠ c. Statistically significant differences (P < 0.05; Student’s t-test) between the non-mutated and mutated IRES, for each haplotype, are indicated by different letters: A ≠ B.

Two genomic regions were selected as markers of the breeding values in terms of virus productivity of the different individuals of the HAV quasispecies. The first region, which spans nucleotides 332–762 (Fig. 1a) and covers 75% of the IRES including the polypyrimidine tract 2 (pY2), was chosen since mutations in this region may reflect differences in the translation efficiency^2^,^18^,^19^. The second region, spanning nucleotides 2394–2852 in the VP1 coding region and including 50% of its total length, was selected after analyzing the Relative Codon Deoptimization Index (RCDI). The RCDI estimates the match between the codon usage of a virus *vs* its cell host. The RCDI of the whole capsid coding region of the consensus sequence of the ancestor L0 and transcription shut-off adapted F0.05LA and F0.2LA populations was calculated in 15 codon-sliding 100 codon-windows (Fig. 1b). Two regions (codon positions 361–475 and 556–715) showed a RCDI value lower than the average, which is an indication of a more optimized codon usage, but only in the second of these regions the F0.05LA and F0.2LA populations showed lower values than that of the ancestor L0 population. Altogether, it indicates that in conditions of cellular shut-off, the 556–715 region of F0.05LA and F0.2LA populations may be more efficiently translated than that of the ancestor due to an optimized codon usage.

### Genomic selection of populations adapted to different degrees of cellular transcription shut-off

Deep-sequencing analyses of the selected regions in the IRES (nucleotides 332–762) and the VP1 coding region (nucleotides 2394–2852) of the F0.05LA and F0.2LA populations was performed and the different sequences compared to that of the ancestor L0 population.

In the IRES region, 16 haplotypes (named ɸ1 to ɸ16) were detected. All of them were present in population F0.05LA and 4 in population F0.2LA (Fig. 1c and Supplementary Table 1). A total of 7 mutations were found in the form of single, double or triple mutants (Supplementary Table 1). The most common mutation was U359C (37.5% of haplotypes), followed by U726C (31.25%) and U590C (25%).

In the VP1 region a total of 13 haplotypes (named λ1 to λ13) were detected (Fig. 1d and Supplementary Table 2). The most abundant haplotypes were λ4, λ5 and λ2 and λ4 and λ2 in F0.05LA and F0.2LA populations, respectively; 8 (λ1, λ3, λ5, λ8, λ9, λ10, λ11 and λ12) and 3 (λ6, λ7 and λ13) haplotypes were exclusive of F0.05LA and F0.2LA populations, respectively. A total of 7 mutations were present in the different haplotypes compared to λ1, which coincides with the consensus sequence of L0 population. The most abundant codon replacements were I85V, L123F and I146V which existed as single, double, triple or even quadruple mutants, when combined with other minor mutations.

The Mfold algorithm was used to elucidate whether mutations found in the IRES haplotypes may influence the RNA secondary structure. Only in the case of the U590C substitution an alternative structure was predicted, which was characterized by the formation of a multibranch loop connecting IRES domains IV and V (Fig. 1e). Although the U726C replacement is not expected to change the RNA structure, this position has been predicted to interact with the 18S rRNA^19^. Additionally, it is located in the pY2 of the IRES which is longer in HAV and maybe related to its slow translation rate^20^. The alternative structure was common to all haplotypes bearing the U590C replacement, even in the presence of additional mutations. Thus, haplotypes ɸ5, ɸ10, ɸ13 and ɸ16 bear an IRES with a potentially different activity.

On the other hand, mutations present in VP1 haplotypes λ2-λ6, λ8, λ9, and λ11-λ13 induced reductions in their RCDI compared with λ1 (Fig. 1f and Supplementary Table 2), which may associate with an increase of the efficiency of translation in conditions of cellular shut-off.

A functional analysis of the impact of the detected mutations in the IRES activity and on the translation efficiency was performed. First, the mutations born in haplotypes ɸ3 (U359C; most frequent), ɸ4 (A513G; negative control), ɸ5 (U590C; alternative IRES structure), ɸ7 (U726C; mutation in pY2), ɸ10 (U590C and U726C) and ɸ16 (U359C, U590C and U726C) were introduced in the G1RL0 bicistronic vector, a modified version of the G1RC^21^ plasmid, in which the translation of the renilla luciferase gene (*RLuc*) is cap-dependent and that of the firefly luciferase gene (*FLuc*) is dependent on the HAV IRES (Supplementary Fig. 1). Replacements U590C (ɸ5 haplotype), U359C (ɸ3 haplotype) and A513G (ɸ4 haplotype) by themselves did not induce a significant change of the baseline IRES activity (L0) in FRhK-4 cells, and although mutation U726C (ɸ7 haplotype) did show an increase, it was not statistically significant (Fig. 1g). In contrast, the double mutant U590C and U726C (ɸ10 haplotype) and, particularly, the triple mutant U359C, U590C and U726C (ɸ16 haplotype) did show statistically significant (P < 0.001) increases of the IRES activity (Fig. 1g). Second, to test the influence of the codon changes in the efficiency of translation, the VP1 fragment corresponding to haplotypes λ1 (baseline RCDI), λ2, λ4, λ5 and λ6 (with lower RCDI than λ1) was introduced in the G1RCMsKp vector (a modified version of the G1RC to incorporate *Msc*I and *Kpn*I single cloning sites, see Supplementary Fig. 1), under the control of the wildtype HAV IRES, and just before the *FLuc* gene. The FLuc/RLuc ratio in FRhK-4 cells in the presence of 0.05 μg/ml of actinomycin D (AMD) was used as an indication of the efficiency of translation in a balanced situation between transcription shut-off and cell viability^6^. Under this condition, haplotype λ5 was the only one showing a significantly higher (P < 0.04) ratio than λ1 (Fig. 1h). Finally, the three mutations resulting in a more active IRES were introduced in each of the constructs bearing the VP1 fragments in order to confirm the critical role of codon usage on translation efficiency. Indeed an impressive rise of the IRES directed translation was observed in the particular case of the haplotype λ5 but not in the rest of haplotypes, which remained unchanged (Fig. 1h). These results indicate the optimal combination of codons in λ5, particularly, the absence of the mutation ATC → GTC at position 85 and the presence of the mutation TTG → TTC at position 123 (Supplementary Table 2).

In conclusion, population F0.05LA contained individuals with a more active IRES, and with an optimal combination of codons in the VP1 region. Ideally, a 0.72% of individuals could correspond to the combination ɸ16-λ5, but if this was indeed the case, how could they be rescued?

### Selection of ɸ16-λ5 haplotype through molecular breeding between genomically selected populations

**Figure 2:**
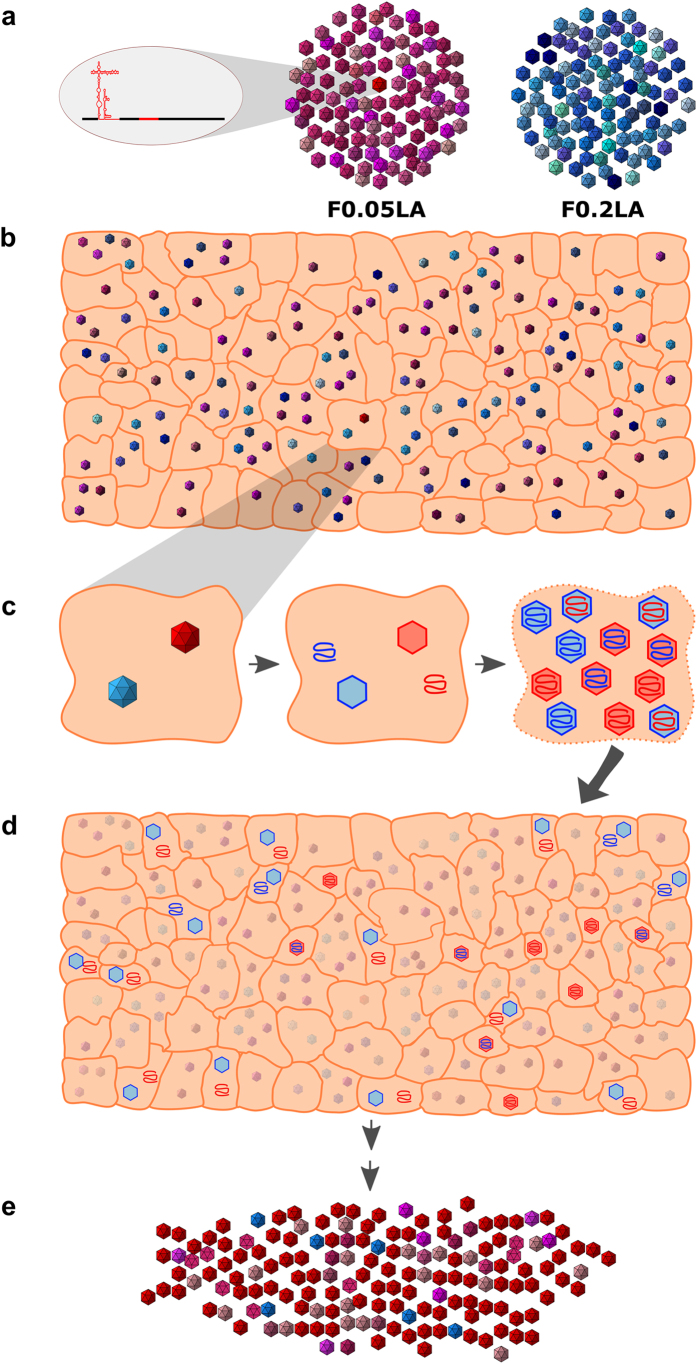
Schematic representation of the process of genomic selection and molecular breeding. (**a**) Viral quasiespecies may contain low frequency high-fitness individuals suppressed by the ensemble of mutants (as the amplified genome from F0.05LA quasiespecies representing a ɸ16-λ5 individual). (**b**) Molecular breeding of quasispecies may be used to rescue these high-fitness variants. Using a moderate MOI, a proportion of cells will remain uninfected while another proportion will be coinfected with more than one virus particle. (**c**) In cells coinfected with two different colonizer viruses, a synchronization of their replicative cycles may occur. In our case, coinfection with F0.2LA and ɸ16-λ5 colonizer individuals, showing fast uncoating time and effective IRES/fast translation, respectively, may result in the emergence of fast chimeric viruses with interchanged capsids and genomes. (**d**) Some of these fast chimeric particles will be able to efficiently infect new cells. (**e**) Finally, the best competitor between both colonizers will be selected (ɸ16-λ5 individuals).

**Figure 3:**
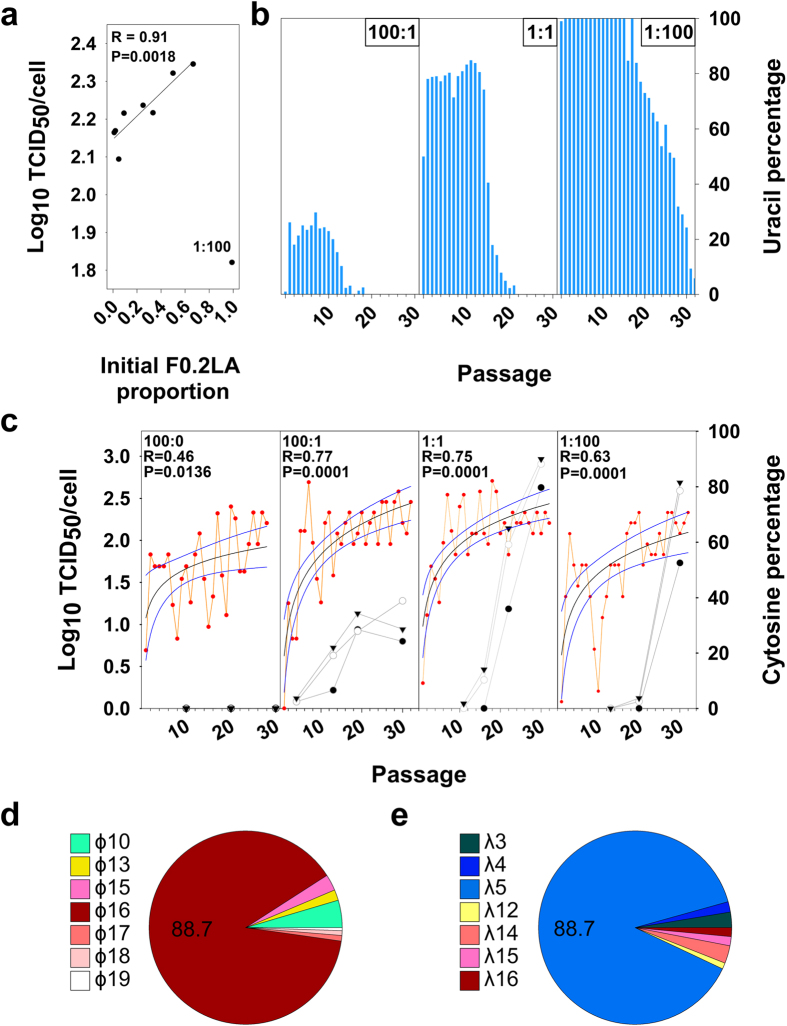
Selection of ɸ16-λ5 haplotype through molecular breeding between F0.05LA and F0.2LA populations. (**a**) Correlation between the initial proportion of F0.2LA population and the virus titers produced. R is the correlation coefficient and P the level of significance. (**b**) Evolution of a genetic marker of F0.2LA population (1298U in VP0 gene) over passages in the molecular breeding experiments 100:1, 1:1 and 1:100. (**c**) Evolution of virus production and emergence of U359C (●), U590C (○) and U726C (▼) IRES replacements over passages in the molecular breeding experiments 100:1, 1:1 and 1:100. A non-linear logarithmic regression between virus production per cell and passages is shown; the black line represents the regression line and the blue lines represent the 95% confidence levels. R is the correlation coefficient and P the level of significance. (**d**) Proportion of haplotypes detected in the IRES fragment of the p30 of the 1:1 (F0.05LA:F0.2LA) breed. (**e**) Proportion of haplotypes detected in the VP1 fragment of the p30 of the 1:1 (F0.05LA:F0.2LA) breed.

The molecular features of ɸ16-λ5 individuals correlate with an efficient colonizer (fast due to a more active IRES) and competitor (ease to obtain translation resources thanks to an optimized codon usage) dual phenotype, which is predicted to render high-fitness individuals. However, these high-fitness individuals are likely suppressed by the ensemble of mutants resulting in a very low frequency. To rescue this variant, a low MOI (to ensure a fraction of initially uninfected cells) and a high population size (to ensure the infection with minority mutants) is required, which implies infection of a large number of cell monolayers. Alternatively, the quasispecies dynamics could be forced by molecularly breeding populations F0.05LA and F0.2LA. This latter population has a shorter uncoating time and higher specific infectivity^13^. Thus, using small cell monolayer formats (24-well plates) with a MOI of 1–2 (theoretically resulting in 37–14% uninfected cells and 26–59% coinfected cells, respectively^22^) some cells may be coinfected with F0.2LA and ɸ16-λ5 individuals, and fast chimeric viruses with interchanged capsids and genomes, able to efficiently infect new cells are expected (Fig. 2). To prove this strategy, different F0.05LA:F0.2LA mixtures (100:1, 50:1. 20:1, 10:1, 3:1, 2:1, 1:1, 1:2 and 1:100) were serially passaged 20 times in FRhK-4 cells and the average production of infectious titers, between passage 6 and 20 measured (Fig. 3a). A strong correlation (R = 0.91) was found between the initial proportion of F0.2LA population and the virus titers produced. From an initial proportion of 1% to 66% there was a statistically significant (P < 0.05) increase of virus production; only the 1:100 mixture behaved as an outlier (Fig. 3a). These results suggested that population F0.2LA indeed contributed in rescuing fast-growing haplotypes. To corroborate this hypothesis we analyzed the consensus sequence over passages in the 100:1, 1:1 and 1:100 breeds looking for the relative proportion of F0.2LA population (Fig. 3b), the emergence of ɸ16 IRES replacements and the evolution of virus production in comparison to population F0.05LA alone (Fig. 3c). Initially, F0.2LA population showed an advantage over F0.05LA population, but later on F0.05LA completely outcompeted F0.2LA (Fig. 3b). In all three breeds, virus production after the appearance of IRES mutations was significantly (P < 0.05) higher than that of the parental populations (Supplementary Table 3). Although the emergence of these mutations always required the presence of population F0.2LA, the time they emerged and the way they increased differed in each breed (Fig. 3c). While first appearance correlated with the initial proportion of F0.05LA population, their increase correlated with the initial proportion of F0.2LA (Fig. 3c). The highest likelihood of coinfection of F0.2LA and ɸ16 individuals occurred in the 1:1 mixture and was of only 0.18% (Supplementary Fig. 2). However, if ɸ16 mutations actually confer a fitness advantage they may be further selected, as indeed happened (Fig. 3c). Although we cannot rule out the possibility that serial passaging of population F0.05LA alone could allow the isolation of ɸ16 individuals too, many more passages would have been required than by using the breeding process with population F0.2LA. In fact, after 30 serial passages of F0.05LA alone none of the IRES mutations were detected (Fig. 3c).

Deep-sequencing of the two regions used in the genomic selection of parental populations was performed in passage 30 of the 1:1 breed, revealing that the dominant haplotypes were, indeed, ɸ16 and λ5 (Fig. 3d,e). Remarkably, both haplotypes were found at a proportion of 89% suggesting that they were linked together in the same molecule. Interestingly, some double IRES mutants from parental F0.05LA population were present too, and some new haplotypes originated in the quasispecies (Fig. 3d and Supplementary Table 1). Similarly, in the VP1 region, some new haplotypes appeared as well (Fig. 3e and Supplementary Table 2). Although it cannot be completely dismissed that the double and triple mutants in the parental populations could be the result of artefactual recombination events during the PCR amplification, it is however remarkable that in passage 30 of the 1:1 breed, most of the individuals were double and triple mutants in the absence of the corresponding single mutants, indicating their real occurrence in the population.

The consensus sequence of the complete genome of passage 30 of breed 1:1 was obtained. All ɸ16-λ5 defining mutations were fixed in the consensus sequence. All nonsynonymous mutations detected were present in the consensus sequence of either the parental or the ancestral types.

In summary, the moderate MOI of the first passage allowed the co-infection of colonizers (F0.2LA and ɸ16-λ5 individuals) resulting in the production of some chimeric viruses (F0.2LA-derived capsids containing ɸ16-λ5 genomes) which efficiently infected new cells, together with F0.2LA and ɸ16-λ5 native virions. In the next passage, at low MOI, colonizers were amplified (Fig. 2). Later on, ɸ16-λ5 individuals outcompeted F0.2LA individuals because they behave as competitors in conditions of high MOI (Fig. 3c).

### Features of the fast-growing population: HM175-HP

**Figure 4:**
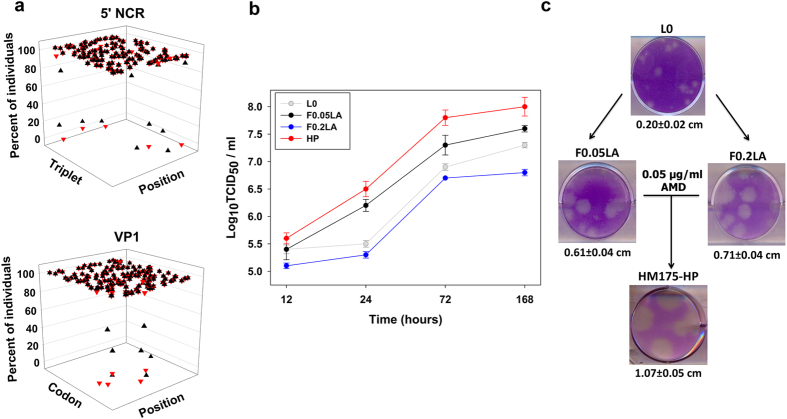
Genomic and biological features of the HM175-HP population. (**a**) Mutation landscape, of the 5′NCR and VP1 fragments under study, of populations F0.05LA (black) and HM175-HP (red). In the 5′NCR 3D plot, the X axis represents the 130 triplets contained in the fragment under analysis, the Y axis represents the 64 possible triplets and the Z axis the frequencies in each population. In the VP1 3D plot, the X axis represents the 139 codons contained in the fragment under analysis, the Y axis represents the 61 coding codons and the Z axis the frequencies in each population. (**b**) One-step growth curves of the HM175-HP, F0.05LA and F0.2LA (parental types) and L0 (ancestor) populations. Three different experiments, titrated in duplicated, were performed. Figures represent the mean and standard error. (**c**) Plaques of the L0, F0.05LA, F0.2LA and HM175-HP populations.

Small differences in the mutation landscape of the 5′NCR and VP1 fragments under study (Fig. 4a), between passage 30 of breed 1:1 and F0.05LA parental type, were predictive of a remarkable change in fitness as ascertained by one-step growth kinetics (Fig. 4b) and plaque diameters (Fig. 4c). Infectious virus titers produced at passage 30 of breed 1:1 were higher than those of F0.05LA or any other population tested (Fig. 4b). Additionally, its plaque diameter in FRhK-4 cells (over 1 cm) was higher than those of other populations and particularly than that of L0 (Fig. 4c; Supplementary Table 4). Altogether, the results point that passage 30 of breed 1:1 is indeed a fast growing population, hence termed HM175-HP (standing for high productivity).

Regarding the antigenic structure, HM175-HP population showed the same level of recognition with a polyclonal serum than L0 population, but a lower level of recognition with mAbs K34C8 and H7C27 (Supplementary Table 5). No differences between these populations were observed neither in their physical stability, nor in the production of “pseudoenveloped” particles (Supplementary Table 6; Supplementary Fig. 3).

## Discussion

Large-scale production of HAV antigen remains an important challenge. Recombinant strategies to produce high capsid yields have systematically failed^23^,^24^,^25^,^26^, which may be related to a low level of expression due to its highly deoptimized codon usage^6^,^13^. An alternative approach could rely on recoding the capsid genome to make it more suitable for translation^7^; yet, this strategy is unlikely to work since the different parts of the genome co-evolve. Therefore, a codon optimized capsid genome would not be efficiently translated with an inefficient IRES^3^ and without a mechanism to shut down the cellular protein synthesis^4^. Additionally, in HAV the need to control the speed of translation in the capsid coding region seems to play an important role in favoring the use of non-optimal codons^6^, which has also been described in proteins involved in critical functions such as the cell cycle control^27^,^28^,^29^. In such a complicated scenario, we moved ahead by adapting the virus to conditions of artificially-induced cellular shut-off with the aim to prompt a ripple effect on codon usage and IRES activity^13^, and several populations showing a faster growing phenotype were selected^13^.

With the goal of further minimize the replication time and maximize the virus yield, a genomic selection approach was used to identify high-fitness minority variants present in the populations. The analyses of the mutant spectra to recognize minor variants with increased virulence, resistance to antivirals or antibodies has been extensively described^30^,^31^,^32^,^33^,^34^. In contrast, to our knowledge, such kind of analyses aiming at identifying minor variants with good “breeding values” with biotechnological usefulness are nonexistent.

We identified two regions in the HAV genome predictive of its fitness relative to the role the IRES and codon usage may have in the efficiency of translation. After deep-sequencing these regions, minority variants with an anticipated fast-growing and high yield phenotype, or in other words with a good “breeding value”, were detected. High-throughput sequencing technologies provide a detailed view of the composition of a viral population, which is overlooked with the coarse focus provided by the consensus sequence, or the classical analysis of quasispecies^31^,^32^,^35^.

*In vitro* assays of IRES activity^21^ and translation efficiency^36^ of the VP1 region under study suggested that these minor variants could have higher fitness than the dominant mutants. Additionally, following population dynamics principles, these variants were predicted to be better competitors and colonizers than the major variants, although they were numerically suppressed by them^14^. Moreover, they were less mutationally robust than the suppressors (Pérez-Rodríguez, manuscript in preparation), which may also contribute to their suppression^31^. Evolution of RNA viruses occurs through disequilibria of the mutant spectra^32^, and co-infections, a source of genetic uncertainty, profoundly affect fitness of RNA virus populations^37^,^38^. To rescue the minority variants, molecular breeding (co-infection) of their bearing population with a colonizer population was performed to induce such a disequilibrium. Selection of minority mutants has usually been based on serial passages with a low MOI and large population size regime^17^, which minimize genetic drift of minority individuals^39^. However, this latter methodology is very expensive and time-consuming, particularly for HAV, which requires over one week of replication for both, virus production and titration. Mutant rescue with a colonizer represents an innovative cost-effective approach in which, despite using a small population size and moderate-low MOI in the first two passages, genetic drift of minority mutants is avoided through early co-infection/complementation with the colonizer (rescuer). The isolation of the fast-growing individuals present in the F0.05LA parental type through molecular breeding with population F0.2LA took advantage of the colonizer individuals from each population that simultaneously and rapidly replicated in co-infected cells. Later on, the best competitors among all these colonizers were selected. Consequently, ɸ16-λ5 individuals are considered to have a dual colonizer-competitor phenotype.

Using the aforementioned approach, we have selected the population HM175-HP that indeed shows a fast-growing phenotype and renders high virus yields. This population was characterized by having a very efficient IRES combined with an optimized codon usage in the VP1 capsid coding region. Codon usage changes involved many nonsynonymous mutations. In consequence, besides the influence of these codon changes in speeding up translation whenever possible and slowing it down when necessary, it cannot be completely ruled out the possibility that these amino acid replacements may play a role on capsid functions critical for an efficient replication cycle. The antigenic and physical features of this population are similar to those of the parental type. Although recognition by mAbs K34C8 and H7C27 was reduced around 30%, convalescent polyclonal antibody recognition was not altered. It might well be that translation-related folding changes and/or the above mentioned amino acid replacements could partially hide the two epitopes recognized by these mAbs, whereas other epitopes might become more accessible thus balancing the overall recognition. Altogether, population HM175-HP is a cost-effective alternative for the antigen producing industry^40^. Although other fast-growing strains of HAV have been previously described^41^, the one herein characterized is to our knowledge the fastest strain ever documented.

While no clear relationship between the severity of hepatitis A infection and mutations in the IRES has never been reported^21^,^42^, their role in increasing virus replication in cell culture is well documented^43^. However, the replication rise observed in the HM175-HP population has no paragon among the HAV strains, and is due to the combined effect of an efficient IRES with codon usage modifications in a context of cellular shut-off. The genomic fragments of the IRES and VP1 regions used in this study were ideal to predict the IRES activity and codon optimization, respectively, and hence could be regarded as markers of the potential productivity or “breeding values” of minority variants. Genomic selection approaches for the isolation of high-fitness minority mutants may be extrapolated to other challenging slow growing agents.

## Methods

### Computer predictions of IRES structures

The Mfold algorithm^44^ available at the mfold Web Server (http://unafold.rna.albany.edu) was used to estimate the secondary structures of the haplotype-derived IRES. Covariant mutations and single/double stranded RNA regions previously described in the HAV IRES^45^ were included as constraint information. For the visualization of the structures obtained the RNA VARNA software tool^46^ was used (http://varna.lri.fr/).

### Computer analyses of the Relative Codon Deoptimization Index (RCDI)

The Relative Codon Deoptimization Index (RCDI) estimates the match between the codon usages of a virus *vs* its host^36^. Although the populations used throughout this study were grown in kidney cells from Rhesus macaque, due to the very similar codon usage between *Homo sapiens* and *Macaca mulatta*, and particularly because of the higher number of genes used to estimate the human codon usage in the Codon Usage Database (*H. sapiens* n = 93487 and *M. mulatta* n = 1051; http://www.kazusa.or.jp/codon/), we used the human codon usage to avoid any bias. An RCDI = 1 indicates that a gene follows the general human codon frequencies, while RCDI values higher than 1 indicate deviations from this general human usage.

To identify any region in the capsid coding genome with a more optimized codon usage in populations F0.05LA (adapted to 0.05 μg/ml of AMD) and F0.2LA (adapted to 0.20 μg/ml of AMD) compared to the ancestor L0 population, an analysis of the RCDI along the capsid coding genome (consensus sequence) was performed. With this purpose, the RCDI was calculated in 15 codon-sliding 100 codon-windows using the public server (http://genomes.urv.es/CAIcal/RCDI/) and graphically plotted to identify the regions susceptible to predict an increased translation efficiency and hence useful in a genomic selection approach.

### Ultra-Deep PyroSequencing (UDPS)

UDPS of both, IRES and VP1, fragments including sequences from both DNA strands were analyzed in single reactions using a multiplex format based on the use of primers including a universal M13 sequence and a multiplex identifier (MID) sequence^47^. The general procedure is described below.

HAV RNA was extracted from 150 μl cell culture supernatants by NucleoSpin RNA Virus kit (Macherey-Nagel), according to the manufacturer’s instructions, and eluted in 50 μl. Two HAV genomic fragments were selected for UDPS (454 GS-Junior Life Sciences, Roche). The first region, spanning nucleotides 332–762, includes 75% of the IRES and was amplified using the following primers: reverse 5′CACAGGAAACAGCTATGACCGGAAAATACCTTGTCTAGAC3′ and forward 5′GTTGTAAAACGACGGCCAGTTTGGAACGTCACCTTGCAGTG3′. The second region, spanning nucleotides 2394–2852 in the VP1 coding region includes 50% of its total length and was amplified using the following primers: reverse 5′CACAGGAAACAGCTATGACCCAGTGCTCCAGACACAGC3′ and forward 5′GTTGTAAAACGACGGCCAGTAAAGTRCCTGAGACATTTCCTG3′. These primers include a template specific sequence and additionally a M13 universal sequence (underlined above) required in the multiplex UDPS^47^. RT-PCRs were performed using the Expand Reverse Transcriptase (Roche) and the high-fidelity *Pwo* DNA polymerase (Roche) and using 5 μl of RNA. PCR products were purified from agarose gel bands using the High Pure PCR Product Purification Kit (Roche), re-amplified for the addition of the MID signaling and purified again. During all the process, as few amplification cycles as possible were used to avoid recombination events which could give rise to artefactual double and triple mutants. The quality and quantity of the DNA was tested using the BioAnalyzer DNA 1000 LabChip (Agilent) and the PicoGreen assay (Invitrogen) and sequences obtained using the GS-Junior Titanium Sequencing kit.

Computations were made using a previously described pipeline^47^,^48^,^49^. Briefly, the fasta file from the GS-Junior was demultiplexed to obtain a fasta file for each sample and strand. Reads not identified by MID and/or primer were discarded. The allowances were two mismatches in the specific sequence, three on the universal M13 sequence and one on the MID sequence with no indels. Sequences not covering the full amplicon or showing more than 2 Ns or 3 gaps were discarded. Sequences not observed on the forward and reverse strands were discarded. Different haplotypes were identified and their frequencies computed as the number of observed reads excluding those with frequencies below 0.5%.

### Sanger sequencing

Standard Sanger sequencing was performed whenever necessary to confirm site-directed mutagenesis and to detect the proportion of populations F0.05LA and F0.2LA and the emergence of IRES mutations during the follow up of some of the molecular breeding experiments (see below).

The complete genome sequence of the fast-growing population HM175-HP was obtained by sequencing 19 overlapping fragments covering the complete genome. These DNA fragments were amplified by RT-PCR using the primers listed in the Supplementary Table 8.

Sequencing reactions were performed using the BigDye Terminator v3.1. cycle sequencing kit (Applied Biosystems) and the ABI Prism 377 DNA Sequencer.

### *In vitro* assays for the determination of the IRES activity

The G1RC plasmid^21^ (kindly provided by Dr. Anne Marie Roque-Afonso, Laboratoire de Virologie, Hôpital Paul Brousse, Villejuif, France) is a bicistronic vector in which the translation of the *Renilla reniformis* luciferase gene (*RLuc*) is cap-dependent and that of the *Photinus pyralis* (firefly) luciferase gene (*FLuc*) is dependent on the HAV IRES. The IRES of the G1RC vector was modified by site-directed mutagenesis in order to get the L0 genetic background, and the obtained vector identified as G1RL0 (Supplementary Fig. 1 and Supplementary Information).

The mutations found in haplotypes ɸ3 (U359C), ɸ4 (A513G), ɸ5 (U590C), ɸ7 (U726C), ɸ10 (U590C and U726C) and ɸ16 (U359C, U590C and U726C) were individually and sequentially introduced into the G1RL0 vector using the same site-directed mutagenesis procedure (Supplementary Information). The list of all primers used is provided in Supplementary Table 7.

For the analyses of the IRES activity, monolayers of FRhK-4 cells grown in 96 well microtiters were transfected with the different vectors. Vector DNA was resuspended in Opti-MEM I (Thermo Fisher Scientific) at a concentration of 0.01 μg/μl and X-tremeGENE HP DNA Transfection Reagent (Roche) was added at a 4% (v:v) concentration. After incubating for 15 min at room temperature, 25 μl of this suspension was added per well. Cells were incubated for a further 30 min at room temperature and finally 60 μl of post-transfection medium (Opti-MEM I) added. After 24h the bioluminescence activity was measured with the Dual-Glo Luciferase Assay System (Promega) and detected with a luminometer (Lumat LB 9507, Berthold Technologies). Light emission was measured 10 min after addition of each substrate and integrated over a 10-s interval. Three different experiments, including two replicas each, for each haplotype-derived vector were performed. IRES activity was figured as the average and standard error of the FLuc/RLuc ratio of all these experiments. The ratio FLuc/RLuc was used to balance the effect of the transfection efficiency.

### *In vitro* assays for the determination of the translation efficiency

The G1RC plasmid^21^ was also modified to incorporate single restriction sites between the IRES and the *FLuc* gene, thus facilitating the cloning of the VP1 fragments. The restriction sites for the *Msc*I and *Kpn*I enzymes were similarly introduced by using the same site-directed mutagenesis procedure above mentioned (Supplementary Information, Supplementary Fig. 1, and Supplementary Table 7) and the modified vector was identified as G1RCMsKp.

A molecular quasispecies of the VP1 fragment under study in the G1RCMsKp vector was obtained. The VP1 fragment was amplified from the viral populations by RT-PCR (Supplementary Table 7). The reverse primer included a restriction site for the *Kpn*I enzyme. Viral RNA extraction, RT-PCRs and PCR products purification were similarly performed as above described but using 10 μl of a 1/10 diluted RNA in the RT reaction. For the cloning experiments, the G1RCMsKp was digested with the *Kpn*I and *Msc*I restriction enzymes and was treated with the FastAP thermosensitive alkaline phosphatase (Thermo Scientific), and the VP1 amplified fragments were digested with the *Kpn*I enzyme. Vector and fragments were overnight ligated at room temperature using the T4 DNA ligase (Thermo Scientific) and transformed into MegaX DH10B T1 Electrocomp Cells (Invitrogen).

Of all molecular clones obtained those containing the fragments corresponding to haplotypes λ1, λ2, λ4, λ5 and λ6 were tested for the efficiency of translation. Additionally the triple IRES mutations associated with a higher activity (U359C, U590C and U726C) were introduced, individually and sequentially using the same site-directed mutagenesis methodology above mentioned (Supplementary Information and Supplementary Table 7), in each of these clones. After confirmation of the introduction of mutations, by sequencing with vector-derived external primers (Supplementary Table 7), these IRES-mutated constructs were also tested for the efficiency of translation. Transfection experiments and bioluminescence determination was performed as above described. The FLuc/RLuc ratio in FRhK-4 cells in the presence of 0.05 μg/ml of AMD was used as an indication of the efficiency of translation normalized *vs* the transcription shut-off and the efficiency of transfection. Three different experiments, including two replicas each, for each haplotype-derived vector were performed.

### Cells and viruses

Three HAV populations (L0, F0.05LA and F0.2LA) previously characterized^13^ were used throughout this study.

Viruses were grown in FRhK-4 cells in the presence of actinomycin D (AMD, Sigma). AMD at concentrations of 0.05 μg/ml and 0.20 μg/ml was added in the post-infection media to inhibit DNA transcription of FRhK-4 infected cells at levels of around 60–70% and over 90%, when growing F0.05LA and F0.2LA populations, respectively^6^,^13^. Infectious virus titers (TCID_50_) were also obtained in FRhK-4 cell monolayers in the absence of AMD.

Mycoplasma presence in FRhK-4 cells was regularly tested using an in-house PCR.

### Molecular breeding experiments

Co-infections between population F0.05LA and F0.2LA were performed using 24-well plates with an average number of 2.5 × 10^5^ cells per well. Different mixture (F0.05LA:F0.2LA) regimes were assayed including 100:1, 50:1; 20:1, 10:1, 3:1, 2:1, 1:1, 1:2, and 1:100. In all cases the multiplicity of infection (MOI) of the first passage was of 1, with the exception of the mixture 1:1 in which a MOI of 2 was used. In the next passage a low MOI in the range of 0.1–0.5 was used. Subsequent serial infection passages (18 with the exception of breeding mixtures, 100:1, 1:1 and 1:100 in which the number of passages was of 28) were done with no further control of MOI. Passages were done every 7 days using a virus inoculum of 0.1 ml. From each passage, viruses were obtained after lysis of infected cells by three freeze-thawing cycles. Lysed cells were centrifuged at 1700 × g for 5 min and the supernatant centrifuged again at 13000 × g for 5 min. This supernatant was used as virus inoculum in the next passage. All experiments were performed in the presence of 0.05 μg/ml of AMD in the post-infection media.

In the particular breeding mixtures, 100:1, 1:1 and 1:100, a follow up of the proportion of the parental populations F0.05LA and F0.2LA and the emergence of the U359C, U590C and U726C IRES mutations was performed by Sanger sequencing of a VP0 fragment, containing a genetic marker^6^, and the 5′NCR fragment under study, respectively. The list of all primers used is provided in Supplementary Table 8.

### One-step growth curves and plaque assays

One-step growth curves in FRhK-4 cells were comparatively plotted between population HM175-HP and the parental (F0.05LA and F0.2LA) and ancestor (L0) populations. HM175-HP and F0.05LA populations were grown in the presence of 0.05 μg/ml of AMD, F0.2LA population in 0.20 μg/ml and L0 in the absence of the drug. Cell monolayers grown in 6-well dishes were infected with a MOI of 5 and freeze-thawed three times at −80 °C at different times post-infection (12 hours, 1, 3 and 7 days). The lysed suspensions were centrifuged at 4000 × g for 20 min and the supernatant titrated twice in FRhK-4 cells. Three independent experiments were performed with each population.

Plaque assays of the same populations were performed in FRhK-4 cells as previously described^50^, but 0.05 μg/ml of AMD was added to the agarose overlay medium with the exception of population L0 which was assayed in the absence of the drug. At 10 days post-infection monolayers were fixed with 4% formaldehyde and stained with crystal violet.

### Statistical analyses

Statistical differences between the different virus populations regarding IRES activity, translation efficiency, virus production/cell, plaque diameter, monoclonal and polyclonal sera recognition and physical stability were assessed using the Student’s t-test (unpaired two-tailed), after verifying the normality of data with the Kolmogorov-Smirnov test.

Linear and non-linear regression and correlation coefficients (R) were calculated in the molecular breeding experiments using the SigmaPlot version 10 and Microsoft Excel 2010.

## Additional Information

**Accession codes:** GenBank KX088647.

**How to cite this article**: Pérez-Rodríguez, F. J. *et al*. Improving virus production through quasispecies genomic selection and molecular breeding. *Sci. Rep.* **6**, 35962; doi: 10.1038/srep35962 (2016).

**Publisher’s note**: Springer Nature remains neutral with regard to jurisdictional claims in published maps and institutional affiliations.

## Supplementary Material

Supplementary Information
