# Supplementary material for: Improving virus production through quasispecies genomic selection and molecular breeding
Source: Sci Rep. 2016 Nov 3;6:35962. doi: 10.1038/srep35962 (PMC5093897; doi:10.1038/srep35962)
Supplement: Supplementary Information [file srep35962-s1.pdf]

SUPPLEMENTARY INFORMATION

Improving virus production through quasispecies genomic selection and molecular breeding

Francisco J. Pérez-Rodríguez[1,2], Lucía D'Andrea[1,2], Montserrat de Castellarnau[1,2], Maria Isabel Costafreda[1,2,7], Susana Guix[1,2], Enric Ribes[3], Josep Quer[4,5], Josep Gregori[4,5,6], Albert Bosch[1,2] and Rosa M. Pintó[1,2]

[1]Enteric Virus Laboratory, Department of Genetics, Microbiology and Statistics, School of Biology, University of Barcelona, Barcelona, Spain

[2]Enteric Virus Laboratory, Institute of Nutrition and Food Safety, Campus Torribera, University of Barcelona, Santa Coloma de Gramenet, Spain

[3]Enteric Virus Laboratory, Department of Cell Biology, Physiology and Immunology, School of Biology, University of Barcelona, Barcelona, Spain

[4]Liver Unit. Internal Medicine. Lab. Malalties Hepàtiques. Vall d'Hebron Institut Recerca-Hospital Universitari Vall d'Hebron (VHIR-HUVH), Barcelona, Spain.

[5]Centro de Investigación Biomédica en Red (CIBER) de Enfermedades Hepáticas y Digestivas (CIBERehd) del Instituto de Salud Carlos III.

[6]Roche Diagnostics SL, Barcelona, Spain.

[7]Present Address: Food and Drug Administration, Silver Spring MD, USA

F.J.P. and L.D. contributed equally to this work

[*]Correspondence should be addressed to R. M. P. (rpinto@ub.edu)

Supplementary Results

Supplementary Table 1. **Haplotypes in the IRES region from the different populations**. Different haplotypes and frequencies were detected in each population through deep-sequencing of the region spanning nucleotides 332-762 (positions in the HM175 strain GeneBank Accession number M14707). Each haplotype was defined by nucleotide replacements with respect to the consensus sequence of the ancestor L0 population.

**Haplotype**	**Nucleotide Replacement**	**Frequency (%)**		
		**F005LA** (N=6768)	**F02LA** (N=7402)	**HM175- HP** (N=12857)
**ϕ1 (L0, F0.05LA*)**	-	39.1	12.24	0
**ϕ2**	C609U	19.99	3.15	0
**ϕ3**	U359C	10.79	0	0
**ϕ4 (F0.2LA)**	A513G	6.96	80.05	0
**ϕ5**	U590C	4.52	0	0
**ϕ6**	U359C C609U	4.15	0	0
**ϕ7**	U726C	3.47	0	0
**ϕ8**	U561C	1.55	0	0
**ϕ9**	G646A	1.14	0	0
**ϕ10**	U590C U726C	1.58	0	4.58
**ϕ11**	C609U U726C	1.57	0	0
**ϕ12**	A513G C609U	1.37	4.57	0
**ϕ13**	U359C U590C	1.20	0	1.78

**ϕ14**	U359C<br>A513G	1.14	0	0
**ϕ15**	U359C<br>U726C	0.75	0	2.72
**ϕ16**	U359C<br>U590C<br>U726C	0.72	0	88.68
**ϕ17**	U359C<br>C609U<br>U726C	0	0	0.94
**ϕ18**	U359C<br>A513G<br>U590C<br>U726C	0	0	0.79
**ϕ19**	U359C<br>U590C<br>C609U<br>U726C	0	0	0.51

*In brackets the haplotype sequence corresponding to the consensus sequence

**ND: Not detected

Supplementary Table 2. **Haplotypes in the VP1 region from the different populations**. Different haplotypes and frequencies were detected in each population through deep-sequencing of the region spanning nucleotides 2394-2852 (positions in the HM175 strain GeneBank Accession number M14707). Each haplotype was defined by nucleotide replacements with respect to the consensus sequence of the ancestor L0 population.

**Haplotype**	**Codon replacement and frequency of use following the cell codon usage**	**VP1 position (aa)**	**Frequency (%)**			**RCDI***
			**F0.05LA** (N=3120)	**F0.2LA** (N=2860)	**HP** (N=3212)	
**λ1 (L0)****	-	-	0.99	0	0	1.716
**λ2 (F005LA)**	ATC (I) (100 %) → GTC (V) (53 %)<br>ATT (I) (61 %) → GTT (V) (34 %)	85<br>146	21.67	24.27	0	1.704
**λ3**	ATT (I) (61 %) → GTT (V) (34 %)	146	12.02	0	2.58	1.698
**λ4 (F02LA)**	ATC (I) (100 %) → GTC (V) (53 %)<br>TTG (L) (26 %) → TTC (F) (100 %)<br>ATT (I) (61 %) → GTT (V) (34 %)	85<br>123<br>146	36.25	63.57	1.74	1.674
**λ5**	TTG (L) (26 %) → TTC (F) (100 %)<br>ATT (I) (61 %) → GTT (V) (34 %)	123<br>146	25.87	0	88.67	1.668

**λ6**	ATC (I) (100 %) →<br>GTC (V) (53 %)<br>ATT (I) (61 %) →<br>GTT (V) (34 %)<br>GTA (V) (19 %) →<br>GTT (V) (34 %)	85<br><br>146<br><br>162	0	3.99	0	1.689
**λ7**	ATC (I) (100 %) →<br>GTC (V) (53 %)<br>ACC (T) (100 %) →<br>ACT (T) (55 %)<br>ATT (I) (61 %) →<br>GTT (V) (34 %)	85<br><br>116<br><br>146	0	0.91	0	1.721
**λ8**	ATC (I) (100 %) →<br>GTC (V) (53 %)<br>TTG (L) (26 %) →<br>TTC (F) (100 %)<br>ATT (I) (61 %) →<br>GTT (V) (34 %)<br>AGC (S) (100 %) →<br>ACC (T) (100 %)	85<br><br>123<br><br>146<br><br>197	0.58	0	0	1.683
**λ9**	TTG (L) (26 %) →<br>TTC (F) (100 %)<br>ATT (I) (61 %) →<br>GTT (V) (34 %)<br>AGC (S) (100 %) →<br>ACC (T) (100 %)	123<br><br>146<br><br>197	0.64	0	1.74	1.676
**λ10**	ATC (I) (100 %) →<br>GTC (V) (53 %	85	0.64	0	0	1.722

**λ11**	ATC (I) (100 %) →<br>GTC (V) (53 %)<br>ATT (I) (61 %) →<br>GTT (V) (34 %)<br>AGC (S) (100 %) →<br>ACC (T) (100 %)	85<br><br>146<br><br>197	0.61	0	0	1.713
**λ12**	CCT (P) (78 %) →<br>CCC (P) (100 %)<br>ATT (I) (61 %) →<br>GTT (V) (34 %)	110<br><br>146	0.74	0	1.03	1.670
**λ13**	ATC (I) (100 %) →<br>GTC (V) (53 %)<br>TTG (L) (26 %) →<br>TTC (F) (100 %)<br>ATT (I) (61 %) →<br>GTT (V) (34 %)<br>GTA (V) (19 %) →<br>GTT (V) (34 %)	85<br><br>123<br><br>146<br><br>162	0	7.27	0	1.659
**λ14**	TCC (S) (91%) →<br>TCT (S) (77%)<br>TTG (L) (26 %) →<br>TTC (F) (100 %)<br>ATT (I) (61 %) →<br>GTT (V) (34 %)	76<br><br>123<br><br>146	0	0	2,96	1.693
**λ15**	CCT (P) (78 %) →<br>CCC (P) (100 %)<br>TTG (L) (26 %) →<br>TTC (F) (100 %)<br>ATT (I) (61 %) →<br>GTT (V) (34 %)	110<br><br>123<br><br>146	0	0	1.53	1.639

**λ16**	TTG (L) (26 %) → TTC (F) (100 %)<br>ATT (I) (61 %) → GTT (V) (34 %)<br>GCC (A) (100 %) → GTC (V) (53 %)	123<br>146<br>157	0	0	1.49	1.664

*RCDI: Relative Codon Deoptimization Index calculated using the following server: http://genomes.urv.es/CAIcal/RCDI/

**In brackets haplotype sequence corresponding to the consensus sequence

Supplementary Table 3. **Virus production in passages subsequent to the appearance of IRES mutations in the different breeds compared to the F0.05LA and F0.2LA parental populations.** Virus production is expressed as the mean ± standard error of the Log_{10} $TCID_{50}$/cell of 10 passages. Statistically significant differences ($p<0.05$; Student's t-test) between breeds are indicated by different letters: a≠b, a≠c, b≠c, ab=a, ab=b, ab≠c.

	Molecular breeds			Parental types	
	**100:1**	**1:1**	**1:100**	**F0.05LA**	**F0.2LA**
**Production/cell**	2.18 ± 0.06^{ab}	2.22 ± 0.05^{a}	2.09 ± 0.06^{b}	1.73 ± 0.07^{c}	1.64 ± 0.11^{c}

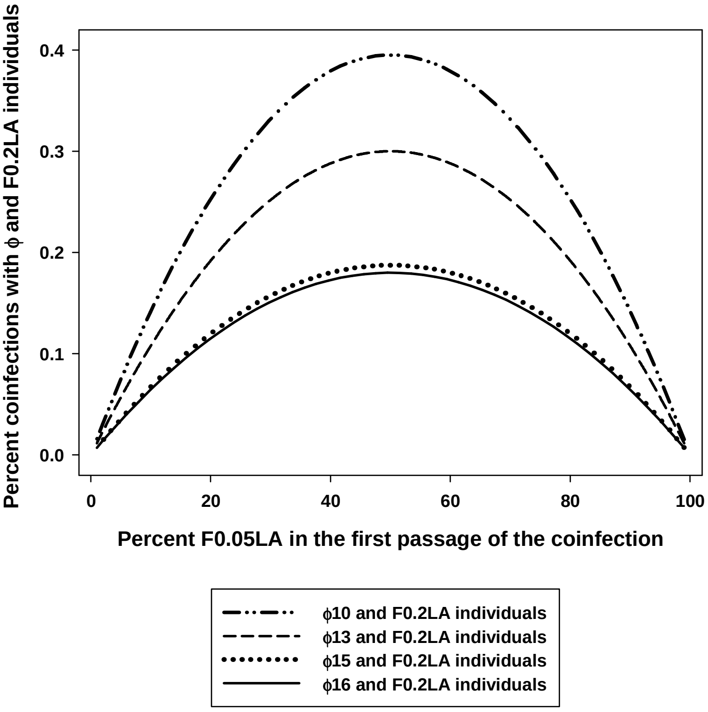

Figure S2. Likelihood of coinfection of different ϕ haplotypes and F0.2LA individuals in theoretical breeding mixtures. F0.2LA population was used to rescue low frequency haplotypes from the F0.05LA through molecular breeding events. The likelihood of rescuing these haplotypes is frequency-dependent. On the one hand the optimal ratio of F0.05LA:F0.2LA is 1:1 (50% of each). On the other, the higher the frequency of a haplotype, ϕ10 (1.58%), ϕ13 (1.20%), ϕ15 (0.75%) and ϕ16 (0.72%), the higher the probability of its rescuing.

Supplementary Table 4. **Diameter of plaques produced by the ancestor and parental type populations and passage 30 (F0.05LA:F0.02LA) breed populations.** Plaque diameter is expressed as the mean ± standard error of 20 plaques in FRhK-4 cells in the presence of 0.05 µg/ml of AMD with the exception of L0 which were measured in the absence of AMD. Statistically significant differences ($p<0.01$; Student's t-test) between breeds, ancestor and parental type populations are indicated by different letters: a≠b, a≠c, b≠c, b=bc, c=b,c .

Populations	Diameter (cm)
**L0**	0.20 ± 0.02[a]
**F0.05LA**	0.61 ± 0.04[b]
**F0.02LA**	0.71 ± 0.04[b,c]
**100:1**	0.90 ± 0.10[b,c]
**1:1 (HM175-HP)**	1.07 ± 0.05[c]
**1:100**	0.95 ± 0.16[c]

Supplementary Table 5. **Antigenic recognition of L0 ancestor and HM175-HP populations.** Recognition of 1 X 10^6 $TCID_{50}$ by two monoclonal antibodies (mAb) against the immunodominant site (K34C8) and the glycophorin A binding site (H7C27) and a polyclonal convalescent serum. Values represent the mean ± standard error of ELISA absorbance readings of three different experiments. Statistically significant differences ($P<0.05$; Student's t-test) between both populations are indicated by different letters: a≠b.

Populations	Recognition by antibodies		
	**mAb K34C8**	**mAb H7C27**	**Polyclonal convalescent serum**
**L0**	0.15 ± 0.01[a]	0.20 ± 0.01[a]	0.19 ± 0.01[a]
**HM175-HP**	0.10 ± 0.01[b]	0.16 ± 0.01[b]	0.17 ± 0.02[a]

Supplementary Table 6. Comparative physical stability of L0 ancestor and HM175-HP populations. Values represent the mean ± standard error of Log_{10} (N_t/N_0) of three different virus stocks, were N_t is the titer after the treatment and N_0 the titer of the same viral stock with no treatment. Statistically significant differences ($p<0.05$; Student's t-test) between both populations are indicated by different letters.

Populations	Stability		
	5 min<br>61ºC	60 min<br>pH 2	240 min<br>1% biliary salts
**L0**	-1.30 ± 0.15[a]	-0.64 ± 0.05[a]	0.12 ± 0.11[a]
**HM175-HP**	-1.28 ± 0.08[a]	-0.46 ± 0.04[a]	0.04 ± 0.04[a]

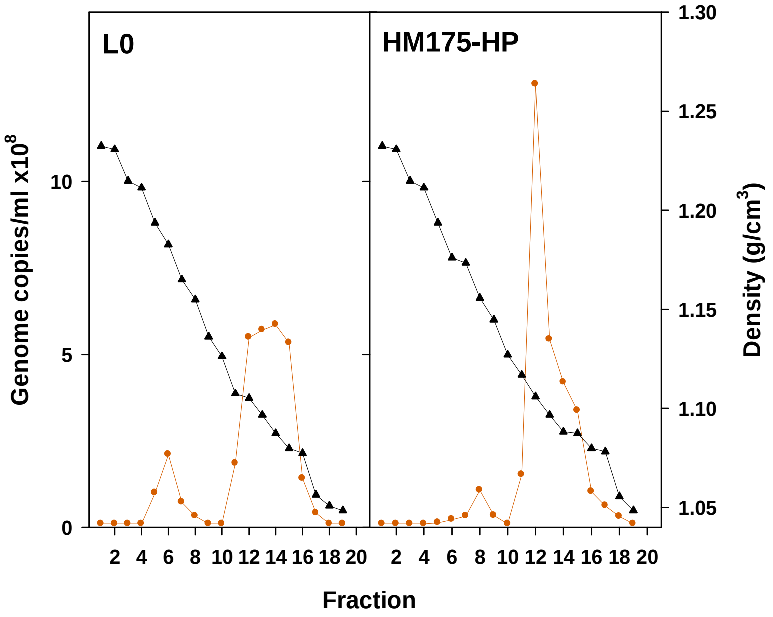

Figure S3. Sucrose-Iodixanol gradients of HAV particles. Supernatants from FRhK-4 cells infected with the L0 ancestor population and the HM175-HP fast growing population were analyzed. No significant differences were observed in the production of "pseudoenveloped" particles. (●) is the HAV genome copy numbers/ml in each fraction; (▲) is the density g/cm^3 of each fraction.

Supplementary Methods

Construction of vectors G1RL0 and G1RCMsKp

For the construction of the G1RL0 vector, five mutations were sequentially introduced into the G1RC[1] vector. Two of them (C140T and A194G) were introduced at once, following a method previously described[2], while the other three (A394G, C473T, C647A) were individually introduced, using the QuikChange Site-Directed Mutagenesis kit (Agilent Technologies). All introduced mutations were confirmed by sequencing using vector-derived external primers. The list of primers is provided in **Supplementary Table 7**.

For the construction of the G1RCMsKp vector, the same site-directed mutagenesis procedure was used to introduce restriction sites for the *Msc*I and *Kpn*I enzymes between the IRES and the *FLuc* gene. All introduced restriction sites were confirmed by sequencing using vector-derived external primers. Primers used are shown in **Supplementary Table 7**.

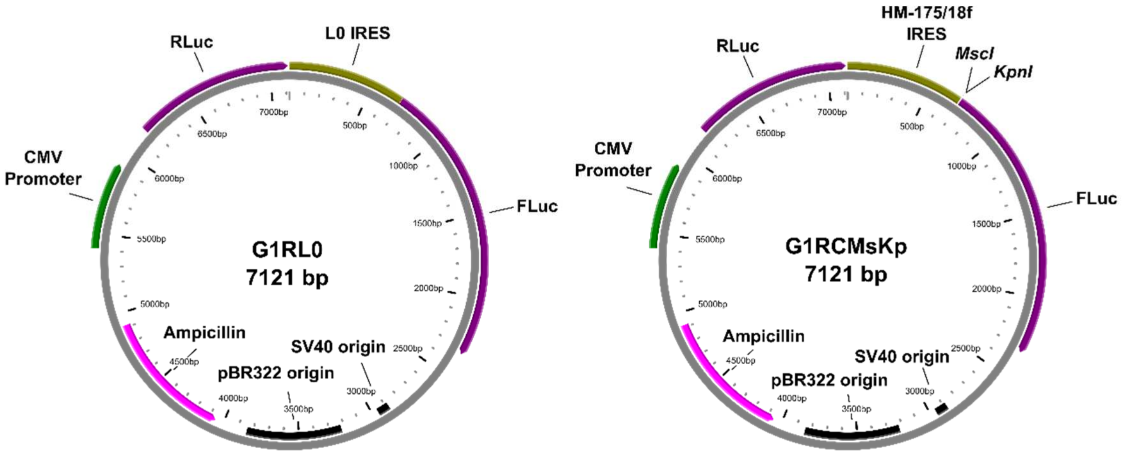

Figure S1. G1RL0 and G1RCMsKp vectors.

Recognition by H7C27 and K34C8 monoclonal antibodies.

Virus stocks used in the analysis of recognition by antibodies were treated with 1% NP-40 for 30 min at 37ºC. Viruses recovered in the supernatants were subjected to three 30-s sonication cycles of at 60 W.

H7C27 mAb recognizes the glycophorin A binding site[3, 4] while K34C8 mAb is directed against the immunodominant site[3]. While H7C27 epitope is present in the protomers, procapsids and capsids, the epitope recognized by the K34C8 mAb is present only in procapsids and capsids[5]. For the recognition with each individual mAb an indirect sandwich ELISA was performed[6], in which particles were captured by a convalescent-phase serum and detected with H7C27 or K34C8 mAbs. All mAbs were used at the highest dilution (1/10000) yielding recognition and were detected using a labeled anti-mouse IgG. An average of 1.5 x 10^6 $TCID_{50}$ per well was used.

Recognition of virus stocks by a polyclonal convalescent serum was also tested. A direct sandwich ELISA was performed in which particles were captured and detected with the same convalescent-phase serum. In the detection step the antibodies from the convalescent-phase serum were labeled and used at the highest dilution (1/1400) yielding recognition.

A cut-off level was established corresponding to the mean ± 3 SD of the unspecific recognition of mock-infected FRhK-4 cell extracts. Three different stocks of each population were tested twice.

Physical Stability.

Treatments at 61ºC for 5 min, pH 2 for 1h at 37ºC and 1% biliary salts for 4h at 37ºC were performed and the resistance of each population evaluated as previously described[7]. To quantify virus decay, a control test of non-treated viruses kept for the same length of time at 37°C was run in parallel. The Log_{10} reduction of the virus titer after each treatment (N_t) compared with the infectious titer of the parallel non-treated virus control (N_0) was figured. Three different stocks for each population were tested and all samples were titrated twice.

Sucrose-Iodixanol gradients

Infected cell culture supernatant fluids were centrifuged at 1,500 x g for 10 minutes at 4°C to pellet any cellular debris and further clarified by centrifuging twice at 10,000 X g for 30 minutes at 4°C. Viruses were further concentrated by ultracentrifugation at 100,000 X g for 1 hour at 4°C. Finally, the resulting pellet was resuspended in 1 ml of phosphate-buffered saline and briefly sonicated, loaded onto a pre-formed 6-50% iodixanol-(OptiPrep, Axis-Shield) sucrose step gradient, and then centrifuged at 205,000 X g for 2 hours and 45 minutes at 4°C using an SW41 Ti rotor in a Beckman Coulter Optima L-90K Ultracentrifuge. Later, approximately 20 fractions of 0.5 ml each were collected from the gradient and the density of each fraction was determined using a refractometer.

RNA from gradient fractions was extracted using the Nucleospin RNA Virus Extraction Kit (Macherey-Nagel) and HAV genome copy numbers were determined by real-time qRT-PCR as previously described[8]. The RNA UltraSense One-Step Quantitative RT-PCR System (Invitrogen) and the

following primers and probe were used in the Mx3000P QPCR System (Stratagene): reverse HAV240 5'GGAGAGCCCTGGAAGAAAG3', forward HAV68 5'TCACCGCCGTTTGCCTAG3' and probe HAV150 6-FAM 5'CCTGAACCTGCAGGAATTAA3'.

Statistical analysis.

Statistical differences between the different virus populations regarding virus production/cell, plaque diameter, monoclonal and polyclonal antibodies recognition and physical stability were assessed using the Student's t-test (two-sided), after verifying the normality of data with the Kolmogorov-Smirnov test.

Supplementary Table 7. List of primers used for: the modification of the vector G1RC, the introduction of IRES mutations and cloning of VP1 fragments. In bold nucleotides involved in site-directed mutagenesis. Underlined *Kpn* I restriction site. Mut primers are those used in the confirmation sequencing.

Primer	Sequence
**G1RC modification into G1RL0**	
C140T A149G+	5’CCCTTTGTTTTG**T**TTGTAAAT**G**TTGATTTGTAAATATTGATTCCTGCAGGTTC3’
C140T A149G-	5’ATCAA**C**ATTTACAA**A**CAAAACAAAGGGAATAGGAAAGGGAAAAGGGAAAGG3’
A394G+	5’CTTTGATCTTCCACAAGGG**G**TAGGCTACGGGTGAAAC3’
A394G-	5’GTTTCACCCGTAGCCTA**C**CCCTTGTGGAAGATCAAAG3’
C473T+	5’GGGTAACAGCGGCGGATATTGG**T**GAGTTGTTAAGACAAAAACC3’
C473T-	5’GGTTTTTGTCTTAACAACTC**A**CCAATATCCGCCGCTGTTACCC3’
C647A+	5’GGGGATCCCTCCATTG**A**CAGCTGGACTGTTC3’
C647A-	5’GAACAGTCCAGCTG**T**CAATGGAGGGATCCCC3’
Mut G1RL0+	5’ATACCTCACCGCCGTTTGC3’
Mut G1RL0-	5’ CCTTATGCAGTTGCTGTCC3’
**G1RC modification into G1RCMsKp**	
MscI+	5’CCTCATTCTTAAATAATAATG**G**CCATGGC**T**GAAGACGCCAAAAAC3’
MscI-	5’GTTTTTGGCGTCTTC**A**GCCATGG**C**CATTATTATTTAAGAATGAGG3’
KpnI+	5’TAATAATGGCC**AGGGTACC**ATGGCTGAAGACGCCAAAAACATAAAG3’
KpnI-	5’GCCAT**GGTACCCT**GGCCATTATTATTTAAGAATGAGGAAAAACCTAAATG3’
Mut MsKp +	5’TCTGCCAAAGACAGGATGTG3’
Mut MsKp -	5’CCTTATGCAGTTGCTCTCC3’
**Incorporation of IRES mutations found in the haplotypes into G1RL0 vector**	
A513G+	5’CGGAGGACTG**G**CTCTCATCCAGTGGATGCATTG3’
A513G-	5’CAATGCATCCACTGGATGAGAG**C**CAGTCCTCCG3’
T590C+	5’CCTCTCTGTGCT**C**GGGGCAAACATCATTTGG3’
T590C-	5’CCAAATGATGTTTGCCCC**G**AGCACAGAGAGG3’
T726C+	5’GGTTTTTCCTCATTCT**C**AAATAATAATGACCATGGCTGAAGACGC3’
T726C-	5’GCGTCTTCAGCCATGGTCATTATTATTT**G**AGAATGAGGAAAAACC3’
T359C+	5’CACCTTGCAGTGTTAACT**C**GGCTTTCATGAATCTCTTTG3’
T359C-	5’CAAAGAGATTCATGAAAGCC**G**AGTTAACACTGCAAGGTG3’
Mut IRES+	5’TTCTGTCTTCTTTCTTCCAGG3’
Mut G1RL0-	5’ CCTTATGCAGTTGCTGTCC3’

**Amplification of the VP1 fragment**	
VP1*Kpn*I-	5’CCCGGTACCTGATTGTTCTGTGACAGACAAATAACAACT3’
VP1CC 5’P+	5’CCTTTCCTGAATTGAAACCTGGAGAATCC3’
**Incorporation of (U359C, U590C, U726C) IRES mutations in VP1-bearing vectors**	
T359C+	5’CACCTTGCAGTGTTAACT**C**GGCTTTCATGAATCTCTTTG3’
T359C-	5’CAAAGAGATTCATGAAAGCC**G**AGTTAACACTGCAAGGTG3’
T590C+alt	5’CCTCTCTGTGCT**C**GGGGCAAACATCATTTGGCCTTAAATGG3’
T590C-alt	5’CCC**G**AGCACAGAGAGGTCTGGAATTAAGCCTAAAGACAGCCC3’
T726C+alt	5’TTTTCCTCATTCT**C**AAATAATAATGGCCTTTCCTGAATTG3’
T726C-alt	5’TATTT**G**AGAATGAGGAAAAACCTAAATGCCCCTGAGTACC3’
Mut λ-IRES+	5’TTCTGTCTTCTTTCTTCCAGG3’
Mut λ-IRES-	5’TTCTCCAGGTTTCAATTCAGG3’

Supplementary Table 8. List of primers used for the analysis of the molecular breeding experiments complete consensus sequence of the HM175-HP population.

Primer Name	Primer Sequence	Position[1]
5NCR1+	5’TGGTGAGGGGACTTGATCC3’	48-66
5NCR1-	5’GGCGTTGAATGGTTTTTGTC3’	503-484
5NCR2+	5’AGGCTACGGGTGAAACCTC3’	396-414
5NCR2-	5’TCTGCCAAAGACAGGATGTG3’	805-786
VP0N+	5’CAGCTGGACTGTTCTTTGGG3’	648-667
VP0N-[1]	5’TCACCAGGAACCATAGCACAG3’	1198-1178
VP0C+	5’TACAATGAGCAGTTTGCTGT3’	1065-1084
VP0C-	5’GCTCTTGCATCTTCATAATTTG3’	1543-1522
VP3N+	5’GGGACAGGAACTTCAGCTTATAC3’	1380-1402
VP3N-	5’AATCTACCTGAATGATATTTGG3’	1861-1840
VP3i+	5’GTTATTCCAGTTGACCCATATT3’	1701-1722
VP3i-	5’TGTATACCTGTTCACTCTGTA3’	2030-2010
VP3C+	5’TGTGCAGTAATGGATATTACAG3’	1938-1959
VP3C-	5’GTTGTTATACCAACTTGGGGA3’	2287-2267
VP1N+	5’AATGTTTATCTTTCAGCAAT3’	2136-2155
VP1N-	5’TCTGACAGCTCCAAGAGCAGTTTT3’	2774-2751
VP1C+2	5’TAGGTCTTGCCGTTGATACT3’	2692-2711
VP1C-2	5’CTTGTGAAAACAGTCCCTTC3’	3243-3224
2A+	5’ATCAGAGGAAGATAAAAGATTT3’	3083-3104
2A-	5’TCTACACTCTGCTATTAATCCA3’	3701-3680
2B+	5’GTCTGAAACGGATTTGTGTT3’	3569-3588
2B-	5’AACCAGTTGGAAAAACTCTG3’	4012-3993
2CN+	5’CCAGAATGATGGAGTTAAGG3’	3970-3989
2CN-	5’-TCTGAAGCCACAGGTTTAGT-3’	4534-4515
2CC+	5’GGCAACCAAAATTTGTAAAC3’	4463-4482
2CC-	5’GAGACCACAACTCCATGAAT3’	4998-4979
3AB+	5’GTTTCATTGATGGATTTGCT3’	4911-4930
3AB-	5’CTTCCTAACCAGTCCTGCTA3’	5324-5305
3CN+	5’ATGGTGTAACTAAGCCCAAG3’	5233-5252
3CN-	5’ATAGGGGTTCCATTTACA3’	5674-5657
3CC+	5’TGGCAACATTAGTGACAA3’	5638-5655
3CC-	5’TTTGGAGACCACATTCAT3’	5999-5982
3CD	5’ATTGATAAGAAAATTGAAAGTCA3’	5925-5947
3CD-	5’AACATCCAAATCAGAACAAT3’	6409-6428
3D+	5’GAAGTTGACCAAAAGAGATT3’	6302-6321
3D-	5’ATGATTCTACCTGCTTCTCT3’	6739-6720
3Db+	5’TTTGATGCTAGTCTTAGTCCATTTA3’	6690-6714
3-	5’AAGAAAGTTCATTTAAACAAATCA3’	7439-7416

[1] positions in the HM175 strain GeneBank Accession number M14707

References

1. Mackiewicz, V. et al. Nucleotide Variability and Translation Efficiency of the 5' Untranslated Region of Hepatitis A Virus: Update from Clinical Isolates Associated with Mild and Severe Hepatitis. *J. Virol.* **84**, 10139-10147 (2010).
2. Liu, H. & Naismith, J.H. An efficient one-step site-directed deletion, insertion, single and multiple-site plasmid mutagenesis protocol. *BMC Biotechnol.* **8**, 91 (2008).
3. Ping, L.H. & Lemon, S.M. Antigenic structure of human hepatitis A virus defined by analysis of escape mutants selected against murine monoclonal antibodies. *J. Virol.* **66**, 2208-2216 (1992).
4. Sánchez, G. et al. Capsid region involved in hepatitis a virus binding to glycophorin A of the erythrocyte membrane. *J. Virol.* **78**, 9807-9813 (2004).
5. Stapleton, J.T. et al. Antigenic and immunogenic properties of recombinant hepatitis A virus 14S and 70S subviral particles. *J. Virol.* **67**, 1080-1085 (1993).
6. Bosch, A. et al. A new continuous epitope of hepatitis A virus. *J. Med. Virol.* **54**, 95-102 (1998).
7. Costafreda, M.I. et al. Hepatitis a virus adaptation to cellular shutoff is driven by dynamic adjustments of codon usage and results in the selection of populations with altered capsids. *J. Virol.* **88**, 5029-5041 (2014).
8. Costafreda, M.I., Bosch, A. & Pintó, R.M. Development, Evaluation, and Standardization of a Real-Time TaqMan Reverse Transcription-PCR Assay for Quantification of Hepatitis A Virus in Clinical and Shellfish Samples. *Appl. Environ. Microbiol.* **72**, 3846-3855 (2006).
